# Oxidative stress and DNA damage in agricultural workers after exposure to pesticides

**DOI:** 10.1186/s12995-020-00290-z

**Published:** 2021-01-07

**Authors:** Caterina Ledda, Emanuele Cannizzaro, Diana Cinà, Vera Filetti, Ermanno Vitale, Gianluca Paravizzini, Concettina Di Naso, Ivo Iavicoli, Venerando Rapisarda

**Affiliations:** 1 Department of Clinical and Experimental Medicine, Occupational Medicine, University of Catania, Via Santa Sofia, 89 - 95123 Catania, Italy; 2 Department of Sciences for Health Promotion and Mother and Child Care, Occupational Medicine, University of Palermo, Palermo, Italy; 3 Clinical Pathology and Clinical Molecular Biology Unit, “Garibaldi Centro” Hospital, ARNAS Garibaldi, Catania, Italy; 4 Clinical Laboratories “Girlando and Paravizzini”, Catania, Italy; 5 Department of Public Health, Occupational Medicine, University of Naples Federico II, Naples, Italy

**Keywords:** Pesticides, Agricultural workers, Biomonitoring, ROS, Cancer, Disease

## Abstract

**Background:**

Recent epidemiological studies on workers describe that exposure to pesticides can induce oxidative stress by increased production of free radicals that can accumulate in the cell and damage biological macromolecules, for example, RNA, DNA, DNA repair proteins and other proteins and/or modify antioxidant defense mechanisms, as well as detoxification and scavenger enzymes. This study aimed to assess oxidative stress and DNA damage among workers exposed to pesticides.

**Methods:**

For this purpose, 52 pesticide exposed workers and 52 organic farmers were enrolled. They were assessed: the pesticide exposure, thiobarbituric acid reactive substances (TBARS), total glutathione (TG), oxidized glutathione levels (GSSG), and 8-oxo-7,8-dihydro-2′-deoxyguanosine (8-oxodG), levels.

**Results:**

Correlation between pesticide exposure was positively associated with high TBARS and 8-oxodG levels (*p* <  0.001). A negative association was founded with TG and GSSG and pesticide exposure.

**Conclusions:**

The present investigation results seem to indicate a mild augment in oxidative stress associated with pesticide exposure, followed by an adaptive response to increase the antioxidant defenses to prevent sustained oxidative adverse effects stress.

## Introduction

Pesticides are a large number of compounds with different targets, chemical structure, and biological effects commonly used for crop protection in agriculture [1].

Pesticides entering the human body through breathing, swallowing, and skin absorption [1, 2]; occupational exposure is characterized by long-term and low-level, cyclical in the seasons [2, 3].

Recent epidemiological studies on workers describe that exposure to pesticides is a risk factor for cancer, diabetes, cardiovascular and neurodegenerative diseases [4–13]. In detail, several investigations reports that exposure to pesticides can induce oxidative stress by increased production of free radicals that can accumulate in the cell, damaging biological macromolecules, for example RNA, DNA, DNA repair proteins and other proteins [14, 15], or modifying antioxidant defense mechanisms, as well as detoxification and scavenger enzymes [16, 17].

The biological mechanisms implicated in protection against intracellular oxidative stress include antioxidant enzymes: superoxide dismutase, catalase, glutathione oxidase (GSSH), and glutathione reductase (GSH) [18].

Some investigations report that oxidative stress and DNA damage caused by pesticide exposure are involved in mechanisms linking pesticide exposure with adverse health effects [19–21].

Oxidative stress plays a critical role in the pathogenesis of neurological, endocrinological, and other metabolic dysfunctions through damaging the antioxidants defense [22].

In the working environment, different pesticides are simultaneously used against pests and weeds; consequently, occupational exposure to multiple pesticides is common [11]. Besides, the pesticide handling mode depends on the task (i.e., mixer, harvester, sprayer,...), the type of pesticide used, and the cultivation in which it will be used [4, 11]. The exposure can happen both in the mixing phase and in the plants; this will also be important to determine the main route of assorbimentom, which in most cases remains in the respiratory and cutaneous [23].

When exposed to multiple pesticides, with each at the level of its respective no observed adverse effect level (NOAEL), a single pesticide produces no toxicity response; instead, probably a combination of multiple pesticides could potentially cause damage to various organ system of the body, possibly also due to their interaction [24, 25].

Moreover, the degree of generated damage depends on the substances’ intrinsic toxicity and individual health status and sensitivity [26].

This study aimed to assess oxidative stress and DNA damage among workers exposed to a mixture of pesticides.

## Materials and methods

### Study design

The study was a case-control study carried out in Ragusa (Sicily, Italy), which has a population of ≅320,000. This is a vastly agricultural district with many people employed in about 25,000 farms producing fruits and vegetables in greenhouses and products derived from animal husbandry. All these activities involve the use of large quantities of pesticides. The main crops are carrots, potatoes, and zucchini in open fields and tomatoes, eggplants, peppers, and zucchini in greenhouses [11].

Fifty-two workers were occupationally exposed to pesticides, and 52 organic farmers were recruited. Exposed workers participating in this study carry out their work with personal protective devices (PPD): gloves, masks, overalls, and protective glasses. Pesticide application was made 5–6 times per week and for 6–7 working hours [11].

Organic farmers (control) did not have any contact with the pesticide, even outside of work.

All subjects recruited for the study were invited during the medical examination of health surveillance carried out by the occupational physician.

Exclusion criteria were diabetes, hypertension, thyroid, liver, kidney, lung, and hematological diseases.

Exposed ones were recruited on a seasonal root (April – August) in the cultivation of greenhouse tomatoes. Table 1 reports the pesticides utilized by exposed workers. It was not possible to detect exposure to biological markers concerning pesticides employed.

**Table 1:** Pesticides exposure

Type of pesticide	Active ingredient	Number of applications	Dose
Fungicides	PropamocarbHydrochloride	1	1 L/ha
	Metalaxyl-M	2	3 L/ha
	Cyproconazole	1	0.05 Kg/ha
Insecticides	Thiamethoxam	1	0.4 Kg/ha
	Deltamathrin	3	0.5 L/ha
	Acrinathrin	1	0.5 L/ha
	Abamectin	1	1 L/ha

A structured questionnaire investigating environmental and occupational risks was administered by trained interviewers to gather accurate data on demographics, medical history, healthcare habits, and pesticide and/or other chemical exposures.

Peripheral blood samples (10 ml/subjects) were collected in Vacutainer tubes with ethylenediaminetetra acetic acid (EDTA) (Vacuette, Greiner Bio-One, Kremsmünster, Austria) were collected. After the collection, the tubes were then centrifuged at 3500 rpm for 10 min, and then the plasma was isolated and stored at − 20 °C until analysis.

Spot urine samples were collected in 10 ml polystyrene disposable urine collection tubes and were frozen at − 20 °C until they were analyzed for creatinine and DNA damage markers.

The Ethics Committee approved the study procedure of Catania University Hospital (Catania, Italy) (n.771/2014), and the written informed consent of all subjects was acquired before their inclusion in the study.

### Assessment of pesticide exposure

It was not possible to use a specific biomarker; in addition, the workers performed the jobs in rotation. For this reason, we have estimated the exposure index.

Exposure deals with the aptitude of a pesticide to cause toxic effects over an extended time, usually after repeated or continuous exposure, which may last for the exposed organism’s entire life. The exposure index of pesticide-exposed agriculture workers that measured the relative levels of chronic occupational exposure to the pesticide was calculated as follows:

$$ \kern0.5em \mathrm{Chronic}\ \mathrm{exposure}\ \mathrm{level}=\kern0.5em \log 10\ \left\{\left[\frac{\mathrm{Y}\ \mathrm{x}\ \mathrm{D}}{\mathrm{age}-18}\right]+1\right\} $$

Y is the number of years of occupational exposure to pesticides and D is the most recent estimate of the number of days of pesticide usage per year. Index values from 0.698 to 2.757 (median to highest value) were classed as high chronic exposure; those from 0.698 to 0.000 (median to lowest value) were classed as low chronic exposure [27].

### Oxidative stress markers assay

The oxidative and nitrosative assessments were conducted through the determination of thiobarbituric acid reactive substances (TBARS), total glutathione (TG) and oxidized glutathione levels (GSSG).

The measure of TBARS is commonly used for determining the lipid peroxide content in plasma; this determination was carried out using the ELISA method as recommended by the supplier (R&D Systems, Inc., McKinleyPlace NE, Minneapolis, USA). For the quantitative analysis of TBARS, 150 μL of serum after acid treatment was mixed with 75 μL of the thiobarbituric acid (TBA) reagent. In the occurrence of heat and acid, malondialdehyde (MDA) reacts with TBA to produce a colored end product that absorbs light at 530–540 nm. The final value was expressed in μM. The inter- and intra-assay coefficients of variations (CV) were 1.3 and 4.4%, respectively.

Glutathione is an essential protector against free radical damage; in this investigation, TG and GSSG were analyzed using the ELISA method that abserve the sulfhydryl group of GSH reacts with 5,5′-dithiobis-2- nitrobenzoic acid to produce a yellow colored 5-thio-2-nitrobenzoic acid that absorbs at 405 nm or 414 nm as indicated by the manufacturer (R&D Systems, Inc., McKinleyPlace NE, Minneapolis, USA).

### DNA damage marker assay

Urine samples were defrosted at room temperature and mixed on a rotary mixer for at least 15 min.

Urinary creatinine concentrations were measured using a fully automated clinical chemistry analyzer (Cobas® 6000 Modular Analyzer, Roche Diagnostics, Basel, Switzerland, Europe); the DNA damage markers concentrations were adjusted based on creatinine levels, which were presented as micrograms per gram (μg/g) of creatinine. All samples showed urinary creatinine concentrations between 0.3 and 3.0 g/L, the range recommended by the World Health Organization (WHO) as a criterion for valid spot urine samples [28].

Concentrations of urinary 8-oxo-7,8-dihydro-2′-deoxyguanosine (8-oxodG) were analyzed using liquid chromatography tandem mass spectrometry (LC-MS/MS) as reported in Ledda et al. [29]. The urine samples were processed and analyzed in duplicates, and the repeatability of the method expressed as the coefficient of variation was 10.5%. The 8-oxodG concentration was calculated as the mean of the two measurements. Urinary 8-oxodG concentrations were adjusted to the average-specific urine gravity (1.015 g/ml) using the formula: 8-oxodG x [(1.015–1)/(measured specific gravity − 1)], as well as to nmol 8-oxodG/mmol creatinine.

### Statistical analysis

Data were summarized as mean ± SD for continuous variables and frequencies for categorical variables. Normality was checked by the Kolmogorov-Smirnov test and homogeneity of variance by Levene’s test. Pearson correlation was used to quantifies the relationship between chronic exposure level and TBARS, TG, GSSG, and 8-oxodG levels.

Data analysis was performed using GraphPad Prism ver.7 (GraphPad Software, Inc. USA).

## Results

The exposed group presented characteristics similar to those not exposed (see Table 2). Mainly, all workers were male and there were no statistically significant differences as to age, BMI, smoking habits, alcohol intake, working-age and sun-light exposure.

**Table 2:** Characteristics of study population expressed as a frequency or mean ± SD

	Exposed n.52	Non exposed n.52	***p***-Value
Gender (male)	52 (100%)	52 (100%)	n.s.
Age (yrs)	33.7 ± 1.7	34.2 ± 1.4	n.s.
BMI (kg/m^2^)	21.8 ± 2.1	22.5 ± 1.8	n.s.
Smokers	16 (31%)	17 (33%)	n.s.
Alcohol consumption (g/day)	17.6 ± 8.5	18.7 ± 7.7	n.s.
Family history of cancer	9 (17%)	13 (25%)	n.s.
Working age (yrs)	6.3 ± 2.1	6.9 ± 1.9	n.s.
Sunlight exposure (h/day)	4.3 ± 1.1	4.5 ± 0.8	n.s.
Exposure duration (yrs)	5.1 ± 0.8	/	**< 0.0001**
Hours of spraying (h/day)	3.7 ± 1.4	/	**< 0.0001**
Chronic exposure level	1.11 ± 0.20	/	**< 0.0001**
TBARS (μmol/L)	1.26 ± 0.44	0.94 ± 0.31	**< 0.0001**
TG (μmol/L)	7.83 ± 1.26	10.03 ± 1.45	**< 0.05**
GSSG (μmol/L)	3.96 ± 0.90	4.50 ± 0.88	**< 0.05**
8-oxodG (nmol/mmol creatinine)	29.71 ± 2.27	11.64 ± 2.74	**< 0.0001**

*n.s.* non-significative

Pesticide exposed workers had been averagely exposed to pesticides for about 3.7 h a day for 5 years and chronic exposure levels equal to 1.11 ± 0.20. Table 2 reports the main sample characteristics.

No worker had signs and symptoms of pathology. The routine blood and urinary examination results were normal and showed no difference with the workers in the control group.

TBARS was higher in pesticides exposed workers (*p* <  0.001), while TG and GSSG were more elevated in organic farmers (*p* <  0.05).

**Fig. 1:**
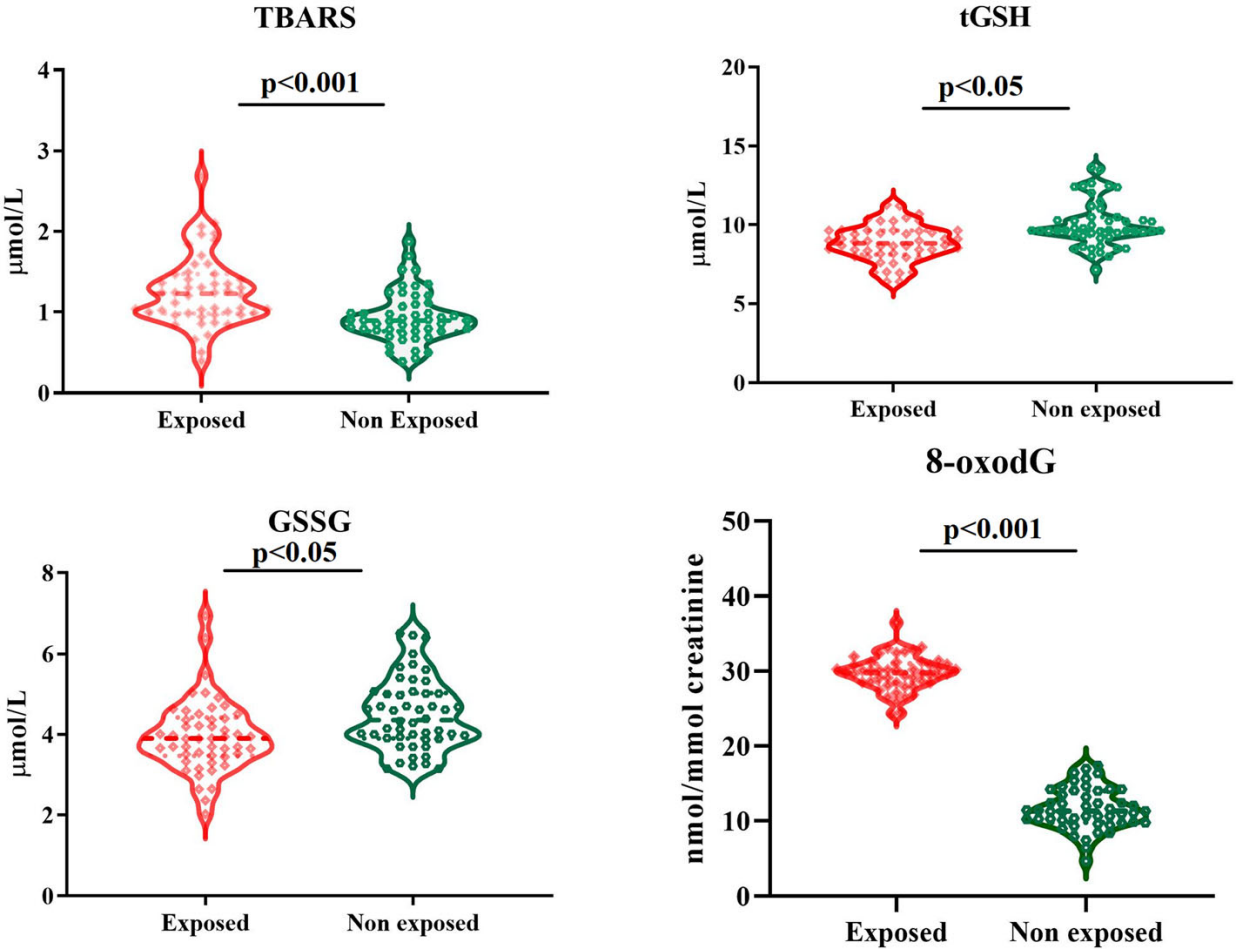
Violin plot of oxidative stress and DNA damage biomarkers

Statistically, the difference was detected in pesticide-exposed workers and organic farmers for DNA damage markers. In Fig. 1 results of oxidative stress and DNA damage graph results.

Correlation between exposure to chronic exposure to the pesticide was positively associated with high TBARS and 8-oxodG levels, r^2^ 0.357 (CI 95% 0.172 to 0.517), and r^2^ 0.648 (CI 95% 0.448 to 0.874) respectively. A negative association was founded with TG and GSSG and chronic pesticide exposure, r^2^–0.425 (CI 95% -0.573 to − 0.249), and r^2^–0.323 (CI 95% -0.489 to − 0.135) respectively.

## Discussion

This study assessed the oxidative stress and DNA damage in workers after exposure (about 5 years) to pesticides. There is increasing experimental support indicating the aptitude of pesticides to induce oxidative stress and sustain recent epidemiological findings [14, 30, 31]. But this investigation falls in the aim of human exposure to low doses of pesticide mixtures, both simultaneously or consecutively, and potential shared toxicological effects, which has been issue to developed scientific interest in the previous years [6, 24, 32].

Under these conditions of exposure, our results have been indicative of a mild oxidative stress and DNA damage in pesticide exposed workers who also appeared to demonstrate an adaptive response to balance for the oxidative discrepancy.

Oxidative stress is believed to be a possible mechanism of toxicity that plays a key role in the toxicological pathway of numerous classes of pesticides, probably due to their metabolism or mitochondrial disruption [33].

Previous investigations have pointed out that various pesticides, including organochlorines, organophosphates, carbamates, and pyrethroids, can induce oxidative stress by generating oxidative molecules or through interfering with the antioxidant defense systems of cells [21, 22].

The excess of highly oxidative molecules is able to attack cell membranes, erythrocytes include, consequencing in lipid peroxidation and additional interference of membrane-dependent progressions [21, 22].

Mainly of pesticides used can interference with oxidative homeostasis across the production of free radicals, lipid peroxidation, and modification in the scavenging enzyme system, causing oxidative stress. This mechanism has been involved in the progress of long-term disorders [2, 16, 20].

TBARS are a direct measure of malondialdehyde (MDA), a reliable biomarker of lipid peroxidation extensively used to evaluate oxidative stress. Augmented TBARS values have been reported in individuals occupationally exposed to different pesticides [2, 16, 20].

In the present investigation, pesticide exposed workers showed higher TBARS levels than organic farmers (*p* <  0.001).

Numerous recent studies underlined the increased TBARS levels in workers exposed to pesticides [34–41]. This shows that enhanced lipid peroxidation correlated with exposure to a different typology of pesticides, either individually or in mixtures; however, the molecular mechanisms are not clear.

The enzyme antioxidant system is the primary defense mechanism against the damage formed by reactive substances [38, 39]. In the present investigation, the levels of TG and GSSG were decreased in pesticide-exposed workers. Physiologically, as the regular levels of oxidative stress are exceeded, extracellular glutathione (GSH) is consumed, and GSSG increases [18]. Therefore, a decrease of TG and GSSG could be considered a loss of prominent defense mechanisms to repair baseline levels of GSH [42, 43]. These conclusions may point out that pesticides may have different aptitudes to induce oxidative molecules. The rate and magnitude of pesticide exposure may trigger or not an adaptive response to turn around the oxidative setting.

Numerous proteins abnormally expressed and/or aberrantly regulated and/or with post-translational alterations have been connected to cancer or neurodegeneration; especially, proteins involved in the DNA damage response [36, 37].

We examined that pesticide exposed workers have a significant increase in DNA damage. These workers confirmed a significant DNA repair activity after exposure to pesticides than not exposed workers. Bacsi et al. [44] reported that decreased levels of 8-oxodG levels resulted in a lower inflammatory response in exposed workers. Consequently, suggesting a function of 8-oxodG in transcriptional activation of proinflammatory mediators in response to oxidative stress [44].

The repair activities induced by 8-oxodG are intricate and modulated by post-translational modifications, as well as phosphorylation and acetylation, furthermore to its interactions with additional repair/nonrepair proteins [44].

Chronic exposure of pesticides is associated with the risk of adverse clinical outcomes, as showed by the tingling, muscle pain, headache, sleep disorder, blurred vision, skin disease, and so forth [45]. The increased level of lipid peroxidation linked with decreased antioxidant defense system, suggesting the enhanced oxidative stress in agriculture workers [45–47].

The assessment of various biomarkers will be useful to detect the adverse health effects among agriculture workers and other individuals exposed to pesticides.

## Conclusion

The results of the present investigation seem to indicate a mild augment in oxidative stress associated with pesticide exposure, followed by an adaptive response to increase the antioxidant defenses. This phenomenon could be essential for preventing the adverse effects of sustained oxidative stress.

Therefore, the evaluation of oxidative stress may be used to advance biomarkers of exposure in workers exposed to pesticides to evaluate early damage.

## Data Availability

The data will be made available to those who make a justified request.
